# NutriPhone: a mobile platform for low-cost point-of-care quantification of vitamin B_12_ concentrations

**DOI:** 10.1038/srep28237

**Published:** 2016-06-15

**Authors:** Seoho Lee, Dakota O’Dell, Jess Hohenstein, Susannah Colt, Saurabh Mehta, David Erickson

**Affiliations:** 1 Sibley School of Mechanical and Aerospace Engineering, Cornell University, Ithaca, NY 14853, USA; 2 Institute for Nutritional Sciences, Global Health, and Technology (INSiGHT), Cornell University, Ithaca, NY 14853, USA; 3 School of Applied & Engineering Physics, Cornell University, Ithaca, NY 14853, USA; 4 Division of Nutritional Sciences, Cornell University, Ithaca, NY 14853, USA

## Abstract

Vitamin B_12_ is necessary for formation of red blood cells, DNA synthesis, neural myelination, brain development, and growth. Vitamin B_12_ deficiency is often asymptomatic early in its course; however, once it manifests, particularly with neurological symptoms, reversal by dietary changes or supplementation becomes less effective. Access to easy, low cost, and personalized nutritional diagnostics could enable individuals to better understand their own deficiencies as well as track the effects of dietary changes. In this work, we present the NutriPhone, a mobile platform for the analysis of blood vitamin B_12_ levels in 15 minutes. The NutriPhone technology comprises of a smartphone accessory, an app, and a competitive-type lateral flow test strip that quantifies vitamin B_12_ levels. To achieve the detection of sub-nmol/L physiological levels of vitamin B_12_, our assay incorporates an innovative “spacer pad” for increasing the duration of the key competitive binding reaction and uses silver amplification of the initial signal. We demonstrate the efficacy of our NutriPhone system by quantifying physiologically relevant levels of vitamin B_12_ and performing human trials where it was used to accurately evaluate blood vitamin B_12_ status of 12 participants from just a drop (~40 μl) of finger prick blood.

Vitamin B_12_ is a water-soluble vitamin that is necessary for DNA synthesis, the metabolism of amino acids and fatty acids^1^ which are required for normal blood formation^2^, cell synthesis^3^, and neurological functions^4^,^5^ in the human body. Low levels of vitamin B_12_ can consequently lead to a wide range of hematologic and neurological abnormalities^6^,^7^. Specifically, poor vitamin B_12_ status has been associated with several acute and chronic conditions including anaemia^7^,^8^, paraesthesia^1^,^9^, and cognitive impairments^10^. Vitamin B_12_ deficiency during pregnancy and early in life is also associated with neural tube defects, poor infant growth and psychomotor function, and impaired brain development^11^. The primary dietary sources of vitamin B_12_ are meats, fish, shellfish, and dairy products and as such the deficiency has been reported to be the highest among populations with predominantly a vegetarian or vegan diet^12^,^13^. In the US, the deficiency rate among vegetarians has been estimated at 60%^14^, while the rate as high as 81% has been reported in India^15^ where there is a high percentage of vegetarians and vegans^16^.

Depending on the severity, underlying cause, and if detected early, the manifestations of vitamin B_12_ deficiency can often be reversed by consuming supplements and/or by changes in diet, as recommended by a healthcare practitioner. In an attempt to reduce the barrier to diagnosis of micronutrient deficiencies, our group has previously developed the vitaAID – vitamin AuNP-based Immunoassay Device – a system for colorimetric quantification of vitamin D levels on a smartphone^17^. In that work a novel gold nanoparticle (AuNP) based immunoassay for vitamin D was developed and anlayzed by our smartphone platform, enabling quantification of serum samples without the need of sophisticated laboratory equipment such as spectrophotometers. However its deployment for point-of-care (POC) applications was limited by the persisting requirements for off-chip blood sample processing, multiple pipetting steps and the non-trivial incubation time (~3 h).

A possible solution to the aforementioned limitations is the adaptation of lateral flow immunoassay principles to diagnose micronutrient deficiencies. Lateral flow assays are widely used in diagnosing numerous diseases^18^,^19^,^20^ and medical conditions^21^,^22^,^23^ in POC settings because they are rapid, simple and produce simple qualitative results that can be interpreted by untrained personnel. Development of lateral flow test for B_12_ however is significantly more challenging due to the extremely low limit of detection that is required. One of the recommended cut-offs for vitamin B_12_ insufficiency is defined in humans by serum vitamin B_12_ below 221 pmol/L^24^ (compared with 50–75 nmol/L 25(OH)D for vitamin D^25^). Traditionally commercial lateral flow assays have lower limits of detection in the tens of nmol/L range^26^.

Here we present a novel POC vitamin B_12_ assessment test, implement it on our “NutriPhone” mobile platform, and demonstrate its performance in a series of human trials. The test uses a finger-stick of blood and comprises of a novel competitive-type lateral flow assay which is able to process whole blood samples, store the necessary reagents, and has built-in flow control directly on the test strip. Controlled and repeatable imaging of the B_12_ test strips with the smartphone platform, and computational image processing via the smartphone app enable the NutriPhone system to provide quantitative information from traditionally non-quantitative lateral flow test strips. As in other smartphone based diagnostics systems, our system inherits the abilities to track changes with time, provide user-free error tracking, and communicate results via e-mail or social networking service^27^,^28^,^29^,^30^,^31^.

In this paper we describe the NutriPhone system and demonstrate how the incorporation of an innovative “spacer pad” for an extended competitive binding interaction, silver enhancement of the initial colorimetric signal^32^, and optical detection via a smartphone platform push the limit-of-detection (LOD) of our B_12_ lateral flow assay into the required range. We show that vitamin B_12_ levels in standard solutions can be accurately quantified in less than 15 min by evaluating the ratio of test to control line signals (T/C ratio). We then validate the performance of our vitamin B_12_ assay in a small trial with human participants.

## Results

### NutriPhone system and vitamin B_12_ test operation

The NutriPhone system consists of: a reusable smartphone accessory, a disposable custom test strip for vitamin B_12_, and a smartphone app. The accessory has been designed to accept test strips and align them to a CMOS camera with an embedded focusing lens (f = 12 mm) in the optical path, which allows for capturing focused images of test strip signals in a compact accessory space. In order to minimize the variability caused by the different external lighting conditions, the accessory relies on two white surface mount LEDs (placed 10 mm on either sides focusing lens, facing the test strip 12 mm away) to uniformly illuminate the front of the test strip while blocking the ambient light entrance. While more LEDs could be adapted (e.g. to illuminate from other angles) to improve the uniformity further, they were compromised for longer accessory operation using two AA batteries at 3 V.

**Figure 1:**
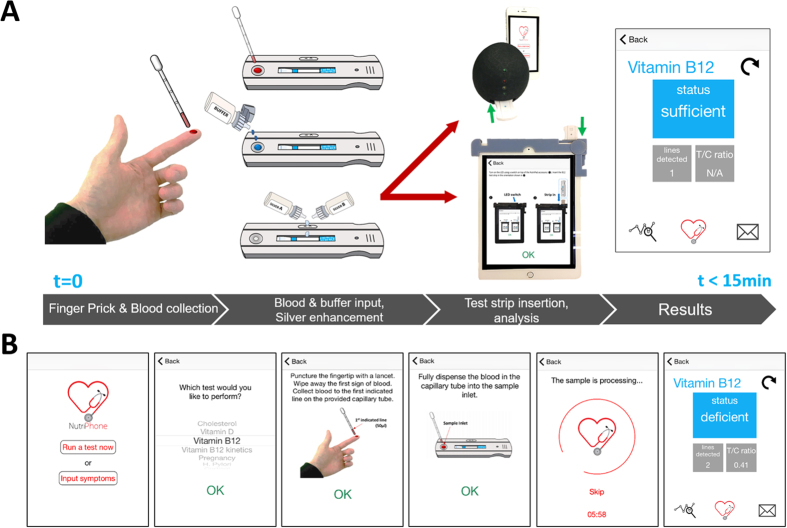
NutriPhone system for PON vitamin B_12_ analysis. **(A)** System overview showing the custom B_12_ test strip that accepts and anlayzes whole blood samples, two versions of the smartphone based accessory that accurately read the test strips, and smartphone app **(B)** NutriPhone app for guiding the users step-by-step through the test protocol and displaying the B_12_ results at the end.

To run a vitamin B_12_ deficiency test in POC settings as shown in Fig. 1a, the user starts the NutriPhone app on his or her smartphone and follows the step-by-step instructions as shown in Fig. 1b. Briefly, the user collects a whole blood sample via a finger prick and applies it directly onto the test strip’s inlet. After allowing for 4 min of incubation, the user initiates the sample flow by applying droplets of the chase buffer from the dropper bottle. The colorimetric signal develops in the subsequent 6 min after which the signals are amplified by applying a droplet of silver enhancement solution from the respective dropper bottles. The user then inserts the test strip into the NutriPhone accessory, and the amplified colorimetric signals are captured by the smartphone camera after 2 min and anlayzed by the smartphone app for the total start-to-results time of less than 15 min.

### Vitamin B_12_ lateral flow assay architecture and principles

**Figure 2:**
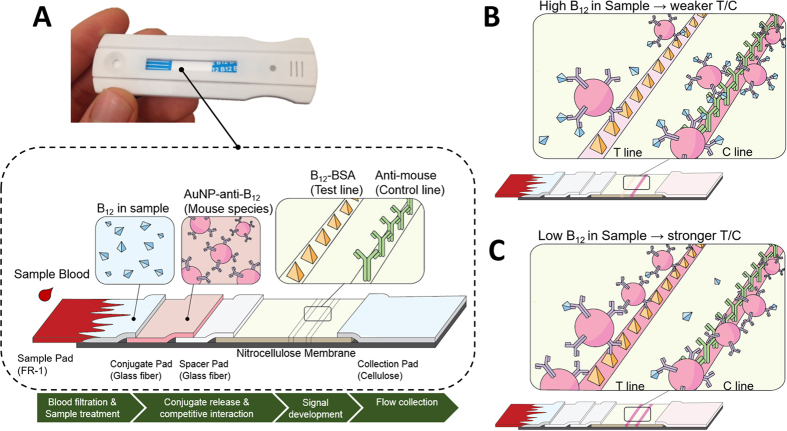
Vitamin B_12_ Lateral Flow Assay. **(A)** Strip image and schematic of the custom B_12_ test strip architecture and components **(B)** Competitive interaction between the sample B_12_ and bound BSA-B_12_ at test line for a limited binding regions on AuNP-anti-B_12_ results in a weak T/C signal intensity for high B_12_ in the sample, and **(C)** strong T/C signal intensity for low B_12_ in the sample.

The custom vitamin B_12_ test strip shown in Fig. 2a was developed as a lateral flow assay which integrates: a whole blood filtration membrane (FR-1), a conjugate pad that dry-stores the AuNP-anti-B_12_ conjugates, a novel spacer pad that allows for longer sample interaction with AuNP-anti-B_12_ conjugates, a nitrocellulose membrane that immobilizes the vitamin B_12_-BSA molecules and secondary antibodies as test and control lines respectively, and a cellulose fibre absorbent pad that collects the waste sample at the end. This architecture represents a competitive-type lateral flow assay which was used here to accommodate the small size of vitamin B_12_ molecules (~1355 g/mol) that prevents their binding to more than one antibody at time^33^,^34^.

When a blood sample is applied onto the sample pad of our vitamin B_12_ assay, the blood cells are filtered and only the plasma proceeds to the conjugate pad and releases the AuNP-anti-B_12_ conjugates that become free to interact with the B_12_ in the sample. The sample to AuNP-anti-B_12_ interaction is the critical step in our assay, where there are limited binding regions on the AuNP-anti-B_12_ and their interaction with the sample B_12_ determines their availability downstream for binding to the BSA-B_12_ on the test line. In conventional lateral flow assays, such interaction occurs over tens of seconds which is too short for an accurate and thorough binding reactions to occur, especially when the target molecules are found in low (i.e. sub-nmol/L) concentrations. The innovation in our NutriPhone B_12_ assay that enables the detection of sub-nmol/L B_12_ has been the addition of the spacer pad between the conjugate pad and the nitrocellulose membrane. This spacer pad effectively prevents the sample—AuNP-anti-B_12_ mixture from flowing onto the nitrocellulose membrane before additionally activated by the user, thereby allowing for a more thorough interaction. In our optimized NutriPhone protocol, the user allows an additional 4 min of interaction time between the sample B_12_ and the AuN-anti-B_12_ before adding chase buffers to complete the test. For samples with high B_12_ levels as shown in Fig. 2b, most of the AuNP-anti-B_12_ conjugates are occupied with B_12_ molecules from the initial sample, resulting in only a subtle change in the colorimetric signal at the test line. Here the preoccupied AuNP-anti-B_12_ that pass the test line without binding are still captured by the secondary antibodies on the control line to exhibit a strong signal, which leads to weak T/C ratios for testing high B_12_ samples. For samples with low B_12_ levels as shown in Fig. 2c, the test line develops an intense colour that reflects the high number of AuNP-anti-B_12_ bound at the test line. This leads a weak control line signal which is indicative of the depleted number of AuNP-anti-B_12_ reaching the control line, and consequently strong T/C ratios are observed for testing low B_12_ samples. For the earlier versions of our B_12_ strips under the same preparation and operating conditions but without the spacer pad incorporation, the T/C ratio remained insensitive to the increase in the sample B_12_ concentrations up until tens of nmol/L which demonstrates the significance of the spacer pad in the B_12_ detection in the sub-nmol/L ranges.

### Vitamin B_12_ quantification in standard solutions

**Figure 3:**
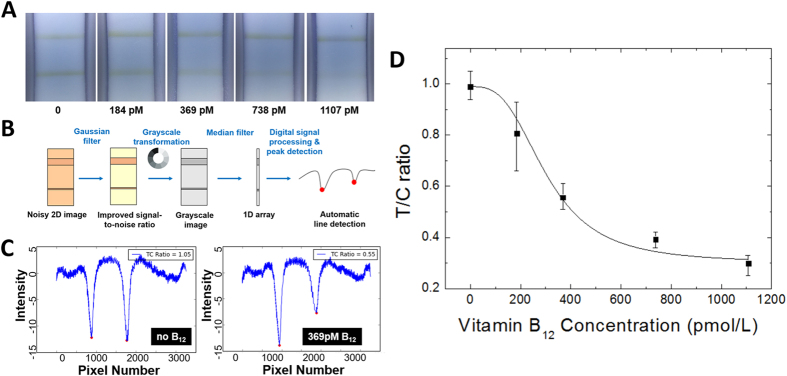
NutriPhone image acquisition and processing for vitamin B_12_ quantification. **(A)** Colorimetric variation of the test and control line regions on the silver enhanced B_12_ lateral flow test strip at different known concentrations of standard vitamin B_12_ samples (**B**) image processing algorithm used by our NutriPhone platform **(C)** Test and control line signals detected by the NutriPhone app as local intensity minima for 0 and 369 pmol/L of B_12_ standard samples **(D)** Calibration curve showing the T/C ratios of the colorimetric signals at different standard B_12_ concentrations: T/C ratio = d + (a–d)/(1+([B_12_]/c)^b), where a = 0.99, b = 3, c = 303.7, and d = 0.3. Four parameter curve was fitted with R^2^ = 0.8776. At each concentration 3 strips were used and the maximum and minimum deviation from the average were shown as error bars.

Once the competitive binding of AuNP-anti-B_12_ conjugates on the test and control lines was performed, the resulting colorimetric signals were silver enhanced and captured by our NutriPhone system. In Fig. 3a, we show the colorimetric change of the test and control lines at different known concentrations of vitamin B_12_ standards. The captured images were then processed by our NutriPhone app based on the algorithm shown in Fig. 3b. Briefly, a noisy 2D image was filtered and converted to the grayscale, followed by a median filtering to reduce the 2D image into a 1D array which simplified our task to a 1D digital signal processing problem. As shown in in Fig. 3c, the test and control regions of highly concentrated AuNP-anti-B_12_ were detected as local minima on the grayscale intensity plot, and the respective peak values could be used to calculate T/C ratios. In Fig. 3d we show that T/C ratios can be correlated to the vitamin B_12_ concentration in the standard solutions. At each concentration, three test strips were used and the maximum lower and upper deviations from the average T/C values were shown. The coefficient of variation of our assay to range from 5.62% at 0 pM of B_12_ to 16.9% at 184 pM of B_12_. We then fitted a four-parameter logistic curve such that [B_12_] = f(T/C). The calibration function was stored and used by the NutriPhone app to predict B_12_ concentrations from the T/C ratios, where the calibration was performed for each batch of test strips in order to account for the batch-to-batch variations.

### Vitamin B_12_ deficiency testing using whole blood samples

**Figure 4:**
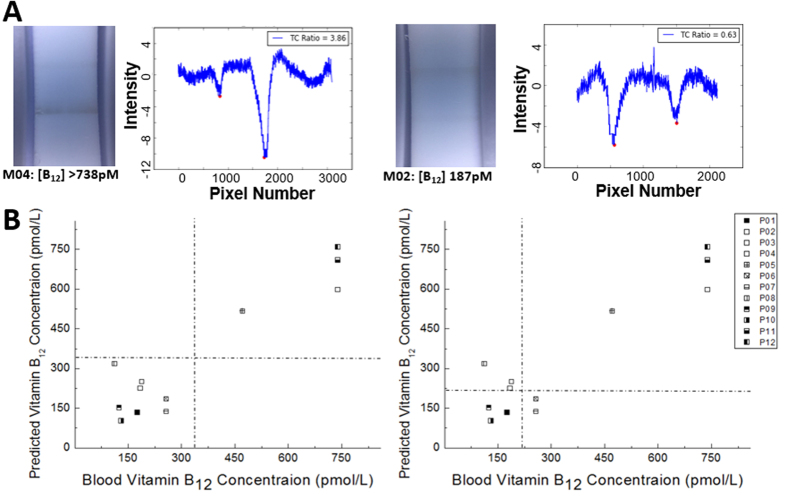
NutriPhone vitamin B_12_ human trials. **(A)** B_12_ test strip images and the corresponding T/C signals as illustrative results from the human trials **(B)** Blood vitamin B_12_ concentrations of 12 human participants as predicted by the NutriPhone system and comparison to the standard method results, where B_12_ deficiency cut-off was set differently at 332 pmol/L (left) and 221 pmol/L (right).

The performance of our NutriPhone system in POC settings was evaluated by using it to diagnose the vitamin B_12_ status of human participants. Here, a single finger prick of blood was collected from each of the 12 participants and used as direct inputs to our NutriPhone B_12_ test strips. In Fig. 4a we show the colorimetric change on the B_12_ test strips for the two participant samples with sufficient (>738 pmol/L B_12_) and deficient (186.7 pmol/L B_12_) levels of vitamin B_12_. As expected for our competitive B_12_ strips, the T/C ratio for a deficient B_12_ case appears to be significantly greater than that for a sufficient B_12_ case, and this difference could be quantified by our NutriPhone app. Fig. 4b demonstrates the vitamin B_12_ levels of the 12 participants as predicted by our NutriPhone B_12_ system based on a calibration curve pre-determined for the tested batch of B_12_ test strips and compares them to the levels as determined by the laboratory standard method (Chemiluminescence Immunoassay on the IMMULITE 2000 Immunoassay system, Siemens Medical Solutions Diagnostics, Deerfield , IL). The comparison shows that our system has a median bias of −0.3%, and a high correlation at +0.93 (p < 0.0001). The NutriPhone system in the current state has a cut-off of 332 pmol/L B_12_, in which it can accurately distinguish eight participants with B_12_ levels less than 332 pmol/L from the other four participants with B_12_ levels greater than 332 pmol/L. The current NutriPhone system shows a reduced sensitivity/specificity when lower cut-offs are imposed, however it maintains a high sensitivity and specificity of 87% and 100% respectively using a 258 pmol/L cut-off. The NutriPhone’s performance for other cut-off concentrations can also be found from the Reciever operating characteristic (ROC) curve in Supplementary Fig. S2.

## Discussion

In this paper we have demonstrated a NutriPhone system which allows for the POC determination of vitamin B_12_ status in under 15 min. This is achieved by developing a competitive-type lateral flow assay for vitamin B_12_ that incorporates a novel spacer pad and produces colorimetric test/control line signals that can be anlayzed by our system to give quantitative T/C ratios, which can then be correlated with sample vitamin B_12_ concentrations. We further validate the system in a human trial where it was used to diagnose vitamin B_12_ status of 12 human participants using a single drop of finger prick blood as the input to the system. Our device can immediately have an impact in some situations that require screening of <369 pmol/L B_12_ samples and B_12_ quantification in the higher concentration region^35^; however we plan to enable better quantification in the future and tune the device to operate with a lower cut-off of 150 pmol/L B_12_, one of the cut-off points used to diagnose vitamin B_12_ deficiency^24^. These results represent a significant step in the development of a POC tool for accurate and rapid quantification of blood vitamin B_12_ levels, with great potential for deployment in the direct consumers market as well as for clinical and community health care settings in developing countries.

In the near future, the performance of the NutriPhone system for B_12_ should be further validated by ensuring sufficient number of replicate measurements per test; for the current prototype version of NutriPhone, the reported performance could suffer from bias due to the low numbers of replicate measurements that we performed. Before the validation, our current prototype device can be improved in terms of both performance and usability. Currently, the quantification of B_12_ levels in the range 0–332 pmol/L of B_12_ is demonstrated only in standard solutions, whereas the reduced performance in whole blood is likely due to its constituents that interfere with our test’s key binding reaction between the sample B_12_ and the AuNP-anti-B_12_. This could be improved by treating our sample pad to include more effective detergents to facilitate the binding interaction, and/or further optimizing the current incubation time after the initial sample application. On the other hand, the limiting step that prevents our vitamin B_12_ assay from being a complete sample-in-answer-out test is the silver enhancement step which additionally requires the user to apply silver enhancer solutions onto the test strip. For better usability by the end users, the next generation B_12_ test strips could dry-store the silver enhancing reagents on-chip as has been demonstrated elsewhere^36^. With these improvements, we hope to extend the human trials in populations with a wider range of B_12_ status to ensure adequate replicates at the lower end of the physiological and the deficient range of vitamin B_12_.

## Methods

### AuNP-anti-B_12_ conjugate pad preparation

The monoclonal anti-vitamin B_12_ IgG produced in mouse (CalBioreagents Inc.) came in >95% purity and was conjugated with 40 nm AuNPs using the InnovaCoat Gold Conjugation Kit (Innova Bioscinces Ltd.). Briefly, 0.23 μg AuNP in freeze dried form was mixed with 1 μg anti-vitamin B_12_ IgG in 0.01 M amine-free phosphate buffer saline (PBS) buffer at pH 7.4. The anti-vitamin B_12_ IgG attached stably to the surface of AuNP via lysine residues during the 15 min incubation and the reaction was terminated by adding 0.1 M tris-buffered saline (TBS) with 0.1% Tween 20. To remove any excess antibodies, 0.01 M TBS with 0.1% Tween 20 was added in 10 times the volume of the conjugate mixture and was centrifuged at 9000 g for 10 min. Upon removal of the supernatants, the final AuNP-anti-B_12_ conjugates were reconstituted in 0.01 M TBS containing 2% bovine serum albumin (BSA) and the final optical density (O.D.) was checked using Spectramax 384 at 530 nm. The conjugates were stored at 4 °C until use.

To prepare the conjugate pads for the B_12_ assay, the AuNP-anti-B_12_ conjugates were first diluted to 0.060 O.D. in the conjugate buffer (2 mM borate buffer with 5% sucrose) which contained the preservative and resolubilization agents^37^. The Glass Fibre Conjugate Pads (EMD Millipore) with 30 cm × 5 mm dimensions were soaked in the diluted conjugate solution for 1 min, followed by drying at 37 °C for 10 h.

### Vitamin B_12_ lateral flow assay preparation

High Flow Plus 180 Membrane Cards (HF180; EMD Millipore) with a 2 mm clear polyester film backing was used as the assay platform, housing the nitrocellulose membrane and the adhesive parts where the conjugate, sample and absorbent pads could be attached. The nitrocellulose portion of HF180 Membrane Cards is 2.5 cm in length and has a nominal capillary flowrate of 45 seconds/cm, which represents the slowest rate offered by the manufacturer and was chosen for our application requiring highly sensitive detection. Before the assembly, the test and control lines were prepared on the nitrocellulose membrane using the Lateral Flow Reagent Dispenser (Claremont Biosolution) to dispense 0.325 mg/ml Vitamin B_12_-BSA conjugate (CalBioreagents Inc.) and 0.75 mg/ml anti-mouse IgG produced in goat (Sigma-Aldrich Co. LLC), respectively. The two lines are separated by 3 mm and uniform line widths of 1 mm could be obtained by operating the Legato 200 Dual Syringe Pump (Claremont Biosolution) at 6.4 μl/min. The membrane cards were subsequently dried for 2 h at 37 °C, then at room temperature overnight. The B_12_ lateral flow assay was assembled into its final form shown in Fig. 1a by first attaching the spacer pad (i.e. untreated Glass Fibre Conjugate Pads with 30 cm × 5 mm dimensions) to the adhesive region of the assay platform below the nitrocellulose membrane with an overlap of 0.5 mm. The AuNP-anti-B_12_ treated conjugate pad was then attached below the spacer pad with 0.5 mm overlap. The FR-1 Membrane (035; MDI Membrane Technologies) with 30 cm × 13.5 mm dimensions was then attached below the AuNP-anti-B_12_ conjugate pad with the 2 mm overlap to serve as the sample pad of the assay. The FR-1 membrane has a thickness of 0.35 mm and whole blood holding capacity of 30 μl/cm^2^. Notably the 5 mm length of the spacer pad was such that the gap created between the nitrocellulose membrane and the conjugate pad is sufficiently long to prevent the sample—AuNP–anti-B_12_ mixture from entering the nitrocellulose membrane at the initial 40 μl sample application. While the 5 mm length can be increased and retain the proper function of the spacer pad, this would necessarily decrease the length of the FR-1 membrane (where the total available length below the nitrocellulose was set by the HF180 platform’s backing length of 20 mm) and thereby decrease the blood holding capacity for the test. As done for the commercial rapid diagnostic tests, a specialized covertape (Kenosha C.V.) with arrows printed to indicate the sample input location, was attached on the top of these membranes and ensured proper contact between the membranes during the sample flow. The Cellulose Fibre Sample Pad (EMD Millipore) was attached above the nitrocellulose membrane with the 2 mm overlap to serve as the absorbent pad of the assay. The assembled assay was cut into individual strips of 4 mm width using a rotary paper trimmer (Dahle). The strips were then kept sealed in a plastic container to prevent contamination from dirt/dust, at room temperature. Also, silica gel desiccants (Dry-Packs™) were added for moisture control, where condensing water vapour could displace the immobilized agents on the nitrocellulose and/or negatively affect the AuNP–anti B_12_ conjugate stability by causing sugar complexes to form^37^,^38^. Storage up to 4 weeks without loss in strip performance has been observed, however longer storage times of up to one year have been widely used for similar, commercial strips (e.g. strips from AccuBioTech Co., Ltd) that use additional control measures (e.g. lamination of pads and membranes) and proprietary additives for lengthened reagent stability.

### NutriPhone test strip protocol and human trials

In a NutriPhone test for vitamin B_12_ deficiency, the use of capillary tubes and dropper bottles allows the users to apply appropriate amounts of key reagents in POC settings. First, vitamin B_12_ samples (Abcam) in the range from 0 to 1107 pmol/L were prepared in standard 1x PBS buffer solutions. To initiate the B_12_ assay, the user collects the sample up to an indicated line on the capillary tube (~40 μl) and dispenses it fully onto the test inlet. This is followed by applying 2 droplets (~40 μl) of the chase buffer (1x TBS with 1% BSA, 1.5% Tween20, 0.1% sodium azide) from the dropper bottle. The addition of the chase buffer allows the sample to fully wet the conjugate pad and begin the sample B_12_ interaction with AuNP-anti-B_12_, while the presence of the spacer pad prevents the sample from entering the nitrocellulose membrane. After waiting for 4 min, the user adds additional 4 droplets (~80 μl) of chase buffer to allow the sample to proceed beyond the spacer pad and complete the flow through the assay by being collected at the absorbent pad upstream. The initial colorimetric signal develops in the subsequent 6 min at which point the user applies a droplet (~20 μl) each from the silver enhancers A and B (Ted Pella Inc.) directly on the test/control regions on the nitrocellulose membrane. The NutriPhone app automatically takes a test strip image after waiting for an additional 3 min for amplification of the initial colorimetric signal, and computes a T/C ratio. Here, using a stop solution to quench the silver enhancement reaction is a viable option, however it would represent an additional step in our testing procedures which is undesirable for the intended point-of-care applications. We therefore did not use a stop solution, and instead computed T/C ratios from the images taken at the same time point. Throughout the procedures, the NutriPhone app incorporates the step-by-step instructions and timers to prevent its users from the common operation errors.

Our human trials were approved by the Institutional Review Board for Human Subjects at Cornell University, and carried out in accordance with their guidelines and regulations. The informed consent was obtained from all participants. In the trials, the participants were finger-pricked for a drop of blood (~40 μL) which was collected using a capillary tube and dispensed onto the inlet of the B_12_ test strip for carrying out the NutriPhone B_12_ test as described in the protocol above. A trained and certified phlebotomist then drew ~5 mL of blood via venipuncture. Following 1 hour incubation at room temperature, serum was separated from whole venous blood by centrifugation at 2000 rpm for 10 min. Serum vitamin B_12_ was measured by chemiluminescence immunoassay on the Immulite 2000 platform (Siemens Healthcare Laboratory Diagnostics). The Siemens vitamin B_12_ kit reports normal reference values between 142–724 pmol/L with a lower limit of detection at 92 pmol/L.

### NuriPhone app and image processing

The majority of the iOS app was written in Objective-C. The image processing algorithm was written in pure C to improve speed and performance. There were no major 3^rd^ party libraries used in the final version of the app; everything was either the default iOS libraries, or written by the authors. After an image was captured by the NutriPhone app, a series of image processing steps as shown in Fig. 3b were performed to improve the limit of detection and accuracy over the conventional visual inspection. First, a 3 × 3 Gaussian filter was applied to the raw image to smooth out some of the noise. The image was then converted to the grayscale, so that the grayscale intensity could be used as a single-channel measurement that distinguishes the background from the colorimetric lines. The grayscale intensity of the coloured control and test lines are lower than the hue of the background, so that the presence of a line could be detected by finding a local minimum in the hue data. After the grayscale conversion, the 2D image was reduced to a 1D array by replacing each row in the 2D image with the median hue value along that row. The median filtering reduces the noise, and the lower dimensionality reduces the process of peak detection to a 1D digital signal processing problem. The local minima corresponding to the test and control lines were located by stepping through the 1D array and storing all points which are at least 10 intensity values below the last inflection point on both sides. The values of local minima were then compared to yield T/C ratios, which were lastly converted into vitamin B_12_ concentrations based on the pre-determined calibration curve, [B_12_] = f(T/C).

## Additional Information

**How to cite this article**: Lee, S. *et al*. NutriPhone: a mobile platform for low-cost point-of-care quantification of vitamin B_12_ concentrations. *Sci. Rep*. **6**, 28237; doi: 10.1038/srep28237 (2016).

## Supplementary Material

Supplementary Information
