# Supplementary material for: NutriPhone: a mobile platform for low-cost point-of-care quantification of vitamin B12 concentrations
Source: Sci Rep. 2016 Jun 15;6:28237. doi: 10.1038/srep28237 (PMC4908584; doi:10.1038/srep28237)
Supplement: Supplementary Information [file srep28237-s1.pdf]

NutriPhone: a mobile platform for low-cost point-of-care quantification of vitamin B_{12} concentrations

Seoho Lee,[1,2] Dakota O'Dell, [2,3] Jess Hohenstein,[1] Susannah Colt,[2,4] Saurabh Mehta*[2,4] and David Erickson*[1,2]

Supplementary Information:

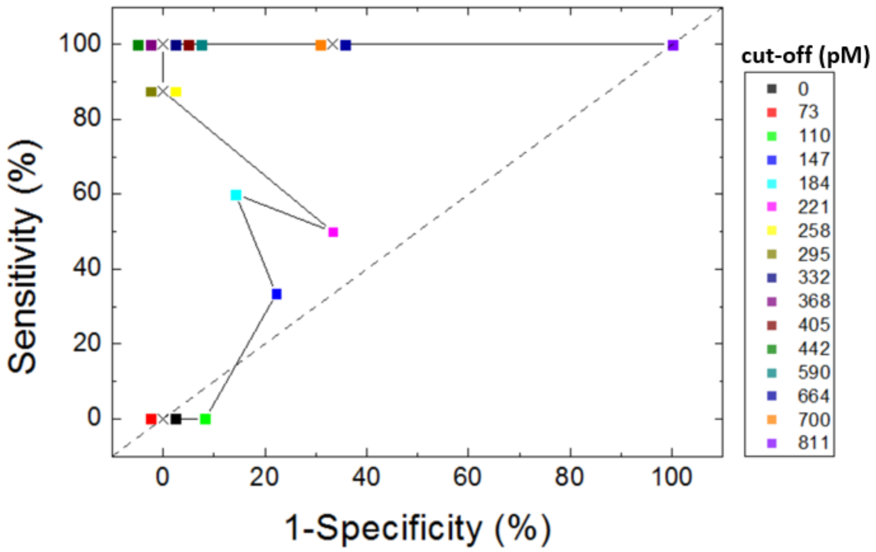

Figure S1: Receive Operating Characteristic (ROC) curve for the NutriPhone human trials. The legend shows the B12 cut-off points, and the dotted line shows the reference line.
